# Dietary Sources and Nutrient Adequacy Potential of Local Foods among Children (6–23 Months) in Urban Slums of West Oromia (Ethiopia)

**DOI:** 10.1155/2020/1787065

**Published:** 2020-11-03

**Authors:** Wondu Garoma Berra

**Affiliations:** ^1^Wollega University, Nutrition Unit, P.O.Box 395, Nekemte, Ethiopia; ^2^Department of Nutrition & Food Hygiene, Hubei Key Laboratory of Food Nutrition and Safety, MOE Key Laboratory of Environment and Health, School of Public Health, Tongji Medical College, Huazhong University of Science & Technology, 13 Hangkong Road, Wuhan 430030, Hubei, China

## Abstract

**Background:**

Identifying the combination of local foods that optimize nutrient intake is challenging. This study addressed how local foods could be rationally combined to provide basic nutritional needs, while limiting the use of commercial foods among children in Ethiopia.

**Methods:**

A cross-sectional survey was carried out to estimate dietary intakes of 396 children (6–23 months of age) using 24-hour recall and WDR. Anthropometrics (weight and height) of the children was taken to calibrate energy and protein requirements to body sizes during ProPAN analysis. Model parameters were defined using dietary and market-survey data. ProPAN (2.0), SAS (9.2), and NutriSurvey for Windows were used for data analysis.

**Results:**

Age-specific optimal combinations of local foods that achieve nutrient adequacy set by the WHO/FAO (≥70% RDA) for 9 nutrients were successfully generated. Overall, the percentage of children consuming ≥ EAR for most nutrients obtained from median servings was 54.3%, 89.9%, 61.8%, 12.9%, 85.6%, 79.7%, and 34.2% for energy, protein, iron, zinc, vitamin A, vitamin C, and calcium, respectively. The percentage of RDA was 46.3% for zinc, 56.7% for vitamin A, 24.3% for vitamin C, and 40% for calcium among infants (6–11 months), whereas the respective percentage of RDA was 78.1% for zinc, 100% for vitamin A, 43.3% for vitamin C, and 50% for calcium in older children (12–23 months of age). However, careful combination of local foods, slightly complimented by commercial foods, has shown substantial improvement in nutrient adequacy, ensuring ≥99% RDA for all target nutrients.

**Conclusions:**

Careful combinations of local foods have the potential to achieve optimum dietary intakes of essential nutrients. However, minimal consideration of commercial foods has been inevitable, especially for infants aged 6–11 months.

## 1. Introduction

Arguably, optimal complementary feeding practice that maximizes uses of locally available food items for children living in developing countries is useful [1, 2]. As a result, the World Health Organization (WHO) often urged developing countries to make use of local resources in order to promote and ensure optimal health, growth, and development of young children.

During complementary feeding period (i.e., 6–23 months of age), breast milk provides substantial part of energy [3, 4]. However, breast milk alone will not meet their nutritional requirements [4, 5]. Breast milk provides only at most 50% infant's high nutrient needs for several micronutrients such as iron, zinc, calcium, and vitamins [4]. Pronounced problem of households' knowledge on food choice was also common in resource-contained environments [5], making diets of young children limited in diversity and nutrients [6].

Despite an array of local foods that could ensure nutrition security in sub-Saharan Africa (SSA) [7], there is lack of knowledge about nutrition for decision-making on selection of locally available nutritious diet and its full cost-benefit analysis [5]. Households in SSA are mostly geared to abetting hunger as a singularity [5]. As a result, malnutrition remains a major problem in SSA [8]. Global Burden of Diseases, Injuries, and Risk Factors Study 2016 (GBD 2016) [9] reported that an estimated 36.6% of children under five were stunted, 8.6% wasted, and 19.5% underweight in SSA in 2015. For example, the Ethiopia Demographic and Health Survey (EDHS) report shows that there is a slight decline in stunting from 44% in 2012 [10], 38% in 2016 [11], and then 36.8% in 2019 [12] among children under five years of age in the country. However, there was little change with underweight (24%) and wasting (10%) of children during 2016 [11] to 21% and 7% in 2019 [12], respectively.

According to evidences [13–15], suboptimal complementary feeding such as poor dietary quality and poor feeding practices is among the main causes of childhood undernutrition in Ethiopia. The national report (EDHS 2016) confirmed that the practices of breastfeeding are almost universal (97%) in Ethiopia. However, only 59% of infants under 6 months are exclusively breastfed [12]. Moreover, child diets are characterized by limited dietary diversity within the country. Only, 7% and 14% of children aged 6–23 months had achieved minimum acceptable diet and access to adequate diverse diet [11], respectively.

Homemade complementary food preparations for child feeding in Ethiopia are predominantly cereal-based (and/or cow milk), and they contain negligible amounts of animal source foods [14]. Moreover, commercial fortified foods are often beyond the reach of the poor [14]. Therefore, optimal complementary feeding practice that maximizes the use of locally available, nutritious, and affordable food items for children living in developing countries could be the best strategy to tackle micronutrient deficiencies [1].

At present, there is a mixed report on the potential value of local foods alone in achieving nutrient adequacy. However, local foods are assumed to be easily accepted by the community, adapted within the normal feeding patterns and cultural food preferences. If optimal combinations of local resources can be achieved successfully, it is possible to assume that nutrient-dense foods could be locally available and accessible sustainably by the target community. A mathematical model that follows local assessment and takes into account cultural variability, feeding practices, and nutrient density profiles of local foods is required to evaluate the energy and nutrient adequacy potential of local foods.

Therefore, the aim of the study was to determine the extent to which local foods could be used, or rationally combined, to provide basic nutritional needs to improve nutrient intakes, while maintaining the livelihood and age-specific dietary patterns observed through dietary assessments for children 6–23 months of age in the study settings.

## 2. Materials and Methods

### 2.1. Study Setting and Design

The study was conducted in urban and periurban slums of Nekemte, West Oromia (Ethiopia). The study area was selected due to diverse agricultural activities, dietary practices, but concern of food security [16]. Furthermore, the subregion is characterized by diverse agricultural activities that are dependent on a single rainy season [13], which is dominantly the lean preharvest period. Two of the six administrative areas of Nekemte were purposively selected due to the estimated large number of young children located in these areas.

The study was a cross-sectional descriptive study in which data on dietary intake and anthropometric measurements were collected during five months (from May to September 2017) to assess dietary consumption patterns of infant and young children (IYC) aged 6–23 months. A market survey was also carried out to determine the cost of commonly consumed local foods per 100 g of edible portion in the study settings.

#### 2.1.1. Subjects and Sampling

A total of 396 children aged 6–23 months were chosen using simple random sampling to estimate population median serving sizes for commonly consumed foods ±10% CI width (assuming 95% CIs and indicator prevalence/median serving size of 50%) [17, 18]. A minimum sample size was covered for each children age-bracket (i.e., 6–8 months (*n* = 101), 9–11 months (*n* = 103), and 12–23 months (*n* = 192)). Nonbreastfed children were ignored in this analysis because the size of children that were not receiving breast milk was too small to be considered as a separate group in LP tools.

Moreover, the inclusion criteria for participation in the present study were children 6–23 months of age who were supposed to be healthy and not suffering from any disability that may affect his/her dietary intakes. As obtaining full records of eligible children living in the study area was difficult, full support from health extension workers (HEWs) was used during the survey. Ethical approval was obtained from the research ethics committee of Wollega University (Ethiopia). And informed consent was also obtained from parents or caregivers of the study participants.

### 2.2. Data Collection

#### 2.2.1. Dietary Intakes

Quantitative dietary data of all sampled children were collected using a single 24-hour recall and weighed diet record (WDR) as described in previous studies [6, 19]. However, duplicate 24-hour recall was administered among 1/8th (*n* = 50) randomly selected subsets of the population on nonconsecutive days to avoid large variations in child's food and liquid intakes from one day to another [18]. In addition to 24-hour recall, the WDR method was also used to collect data on food consumption for 7 days to describe food patterns.

Interview was carried out by four well-trained research assistants jointly with HEWs who were fluent in the local language, and all the lists of foods and beverages consumed by children were recorded and, at the same time, weighed. During the interview, caregivers were asked to list all foods and drinks the child had consumed in the past 24 hours from the child waking up a day before the survey date till the time he/she waking up on the day of the interview. The 24-hour dietary recalls were collected on all days of the week except on Sunday. During the interview, primary caregivers were asked to recall all the foods and beverages consumed by their infants in the past 24 hours.

In WDR methods, all foods and beverages consumed by a child were weighed using the electronic kitchen scale (model EC002, range 5  kg × 1 g). Information on the ingredients and cooking methods for each food item and/or dish was asked. Mothers/caregivers were asked to estimate the amount of recalled foods in local cups or utensils, and the amount was then weighed using real food. All composite foods and dishes were broken down into their individual ingredients. WDR was collected on all days of the week (except Sunday) to account for the effect of most days of the week on dietary intakes of the subjects.

Food consumption data from the WDR were used to estimate the average serving sizes of foods consumed by these children, and data from all dietary assessment methods were used to describe the median and range of food consumption patterns in 7 d.

Portion sizes were estimated in the 24-hour recall by asking mothers/caregivers using real food to show the amount served and the amount left over after feeding. Then, these amounts were weighed on dietary scales and recorded, and the amount consumed by the child was calculated by difference. However, in case food which was eaten by a child or its raw ingredients were not available in the household at the time of the interview, amounts were estimated from similar foods using household units in volume or size, which was to be standardized later and used to calculate the proportion served and eaten by the child.

Accordingly, the 24-hour recall and WDR were analyzed to characterize each food group pattern, create a list of foods consumed by the child, and measure for each food item its average serving size (g/d) and the percentage of children consuming it.

#### 2.2.2. Anthropometrics

Child weight and length were measured (in duplicate) and recorded using standard anthropometric techniques by trained enumerators (HEWs). The tool to collect anthropometric data was essential to provide the average weight of children to calibrate energy (calorie) and protein requirements to body sizes (protein needed per kilogram of body weight) in ProPAN (2.0) analysis. Also, it provides information on the current nutritional status of the target population.

The body weight of children was measured to the nearest 100 g with an electronic scale (Hochoice, model 400-021-6368, range 180 kg, and precision 0.1 kg), while the child was minimally clothed. Recumbent length was measured to the nearest 0.1 cm using the locally modified wooden measuring board with a movable headset. Birth dates were taken from child health cards as the primary source when available and mothers' reports, otherwise.

#### 2.2.3. Market Survey

A ProPAN market cost tool was used to provide a price per 100-gram edible portion of individual foods identified in the 24-hour dietary recall. A market survey was conducted from three big local markets on different days of the week. At most, three prices per food item were recorded with an attempt to include both the highest and the lowest prices for the survey area, and the average cost was entered into ProPAN (2.0) program.

In case of cooked composite dishes, each raw ingredient was weighed, and their costs were summed to obtain the cost for all ingredients. The final cost per 100 g of cooked food or composite dish was then estimated, and later, the cost was linked to the food composition database in ProPAN [20] and LP of the NutriSurvey module [21].

### 2.3. Data Analysis and Interpretations

Dietary and anthropometric data were double-checked and undergone through rigorous cleaning prior to entry and analysis. All data related to dietary, market, and anthropometric measurements were entered into ProPAN 2.0 for analyses. Further analyses were done using SAS 9.2 and NutriSurvey to obtain the optimal output.

#### 2.3.1. Nutritional Status

Anthropometric *Z*-scores for a child were calculated using ProPAN 2.0, based on WHO's 2006 Child Growth Standard [22]. Thus, *Z*-scores for weight-for-age, length-for-age, and weight-for-height of infants and young children surveyed were analyzed. Underweight, stunting, and wasting were defined as a *Z*-score ≤ −2 ± SD.

#### 2.3.2. Actual Food Consumption Pattern and Nutrient Intakes

Quantitative dietary data which were collected using 24-hour recall and WDR and dietary patterns were entered into ProPAN (v2.0) to calculate energy and nutrient intakes. Single-day individual dietary data plus duplicate among 12% of children across 6 days of the week were collected to estimate the dietary patterns. However, mean energy was calculated as ProPAN (v2.0) cannot do adjustments for percent children who meet their requirements if more than one 24-hour recall was administered per child. The actual dietary pattern was derived based on the median amount of food reportedly consumed by participating children, considering foods consumed by ≥ 5% participants of the study.

In the present study, however, only the following nutrients with complete nutrient profiles were considered due to the limited national food composition database. These include energy, protein, fat, carbohydrate, iron, zinc, calcium, vitamin A, and vitamin C. The calculation of energy and nutrient intakes of children from actual intake was based on sources primarily from food composition tables (FCTs) for use in Ethiopia [23]. In addition, this also complemented with FCT data from the United States Department of Agriculture Food Composition Database (USDA) [24] and East Africa, particularly FCT data from Tanzania [25] and Uganda [26]. Moreover, nutrient losses during food preparations were adjusted based on the USDA Table of Nutrient Retention Factors, Release 6 (2007) [27], which is the major source of nutrient retention data for the US and international food composition databases.

A total of 24 food items most frequently consumed among ≥5% of target children were screened and selected from the dietary records. These food items were categorized based on the WHO food groups and culinary usage for children aged 6–23 months [5], namely, grains, roots, and tubers; legumes and nuts; dairy products (milk, yogurt, and cheese); flesh foods (meat, fish, poultry, and liver/organ meats); eggs; vitamin-A-rich fruits and vegetables; and other fruits and vegetables.

Accordingly, energy and nutrient intakes of each child were analyzed using ProPAN (v2.0) to determine the proportion of children below the estimated average requirement (EAR), which is the basis for deriving recommended dietary allowance (RDA). Adequate nutrient intakes of sampled children were assumed if 100% or more of the children meet the proxy EAR as recommended by the Institute of Medicine (IOM) [28]. Nutrient-specific RDA-to-EAR conversion factors of 1.2 for zinc, vitamin C, and calcium and 1.4 for vitamin A were used as recommended by the IOM. However, for protein and iron, conversion factors do not exist. Thus, 66% of the RDA was used as a proxy for the EAR, as previously recommended [29].

#### 2.3.3. Preparations of Model Parameters

The 24-hour dietary recall and WDR were used to generate model parameters, with the preparation of the LP model carried out using ProPAN (v2.0). These parameters have included the energy (kilocalorie) content of each modeled diet (which was equal to the average energy requirement for the target group), lists of foods consumed by ≥ 5% of the target children, and the median, the minimum, and the maximum serving size per day for each foods and (sub) food groups they belong to.

The minimum, median, and maximum limits for serving size were defined at 10th, 50th, and 90th percentile distributions of serving counts reportedly consumed in the datasets. The actual serving size for each food subgroup was defined by the median (50th percentile) serving size for children who consumed food in each age bracket. These parameters were used to set up the models for the analyses in LP of NutriSurvey for Windows [21], and the diets were modeled for a 7-day period.

Accordingly, the LP model has generated optimized solution for the nutritional constraints (energy, fat, protein, carbohydrate, iron, zinc, calcium, vitamin A, and vitamin C). The Food and Agriculture Organization of the United Nations (FAO/WHO 2001) [30] reference for human micronutrient requirement and moderate bioavailability level for both iron (10%) and zinc (30%) was considered in the LP process, as the dietary database in Ethiopia on the bioavailability of these two nutrients was not well established to take more appropriate assumption, which might have possibly changed our results.

Moreover, the quantity of breast milk consumed was not assessed in the dietary survey. As a result, the daily intakes for breast milk were considered as per the WHO reference on complementary feeding of young children in developing countries for children aged 6–8, 9–11, and 12–23 months [31], respectively.

#### 2.3.4. Nutritional Constraints for LP Models

Three sets of constraints were introduced to our LP models to minimize the gap between actual and optimized food intake patterns. The intention was to ensure that the solution selected by a model satisfies each of the specified constraints.

Firstly, food use limits were set to ensure that program models did not exceed the amount of foods that is usually consumed by the target population. These limits were derived from the actual intake patterns for each age group as reported in the WDR. As described earlier, dietary intake of all foods from each food subgroup was required not to exceed the 90th percentile of intake for each age group. Food group and subgroup constraints were defined by the grams of individual foods in each age group. We considered food intakes within the range of 10th and 90th percentiles of the observed intakes for each age group. However, exceptions were made for some food items in the miscellaneous food group (condiments, herbs, spices, and sugar) where the maximum was purposively set at the 50th percentile to avoid excessive intake from this subgroup in the optimized diet.

Secondly, nutritional constraints were included to ensure that the nutritional content of each optimized food intake pattern was equal to or greater than the desired value, which was based on the DRIs for infants and young children feeding practices for developing countries. DRIs for the selected nutrients published by the FAO/WHO for infants and young children [30] were the criterion for determining whether each nutritional goal had been achieved by the optimized food intake pattern.

Energy constraint for moderately breastfed children was also included in the model to ensure that the energy content of the optimized food intake pattern was equal to the estimated energy requirement (EER) for each age group. The energy content of the diets obtained for these age groups is derived from PAHO/WHO guiding principles for complementary feeding of the breastfed child (i.e., 615 kcal for 6–8 months, 686 kcal for 9–11 months, and 894 kcal for 12–23 months).

The energy and nutrient contribution from breast milk were taken into account to ensure that the realistic diets were selected. As the mean breast-milk intakes for the study population were unknown, the published WHO average breast-milk intakes were used in our LP models [31]. These were 674 g/d, 616 g/d, and 549 g/d for children aged 6–8 months, 9–11, months and 12–23 months, respectively.

The minimum protein content of the diet was obtained from the WHO report of complementary feeding of young children in developing countries [31], and it was set at 9.1 g, 9.6 g, and 10.9 g for the age groups 6–8 months, 9–11 months, and 12–23 months, respectively.

The minimum content of fat of the whole diet, including breast milk, was set at 30% of the total energy to be consistent with PAHO/WHO guiding principles on complementary feeding [4], and this represents a minimum fat intake of 20.5 g/d, 22.8 g/d, and 29.8 g/day for the age groups 6–8 months, 9–11 months, and 12–23 months, respectively.

Moreover, the minimum contents of calcium, zinc, iron, vitamin C, and vitamin A included in the present LP models correspond to the FAO/WHO recommended intake [30]. For zinc, we had chosen recommended moderate values for bioavialabilty of 30%, corresponding to the diets of moderate bioavailability, whereas for iron, the corresponding values of bioavailability of 10% were considered.

Finally, the cost constraint was set to ensure that the cost of the optimal formulation was equal to the maximum cost of all diets consumed by the children in each age group.

## 3. Results

### 3.1. Characteristics of the Study Population

The calculated energy (kcal) consumed in 24-hour periods has shown to have huge variability as observed from the standard deviations. As presented in Table 1, the overall mean body weight and calculated average energy (kcal) intakes of children were 9.6 kg and 529.3 kcal/day, respectively.

### 3.2. Actual Food Patterns and Nutrient Intake

A total of 68 food items including condiments were recorded during the dietary survey. However, only 24 food items were selected to be included in LP tools (Table 2) because they were most frequently consumed by ≥ 5% of the target children. The food items were categorized into seven major food groups based on the WHO [32], with little modifications to take into account the only list of foods selected. These are grain, grain products, roots, and tubers; legumes, nuts, and seeds; meats, fish, and eggs; dairy products; vegetables; beverages; and miscellaneous foods.

Considering teff as staple grain foods in Ethiopia, it was expected that teff injera was the most frequently consumed of grain and grain products; nearly, 68% of children rely on it. Other than mitin (a blend of cereal-pulse), the only sole legume-based food item included in our model was pea flour consumed in the form of stew/wot. Often accompanied with other cereal-based foods (mostly injera), pea flour stew was reportedly consumed among 50% of the children. Chicken egg (fried w/fat) was consumed by 19.4%, whereas cow milk was used among 37% of children. Nearly, 56% of children had consumed onion as condiment vegetables. However, the consumption of miscellaneous food items such as added fats, salts, spices, and sugar was relatively higher in the existing actual dietary patterns.

As presented in Table 2, the median serving sizes of injera, pea flour (stew), fried chicken egg, and cow milk varied between 20.0 and 30.0 g/day, 4.2 and 7.7 g/day, 31.0 and 41.5 g/day, and 115.0 and 160.0 g/day, respectively. Moreover, the median number of servings reported for each food item included, when only considering those children who reported ever consuming food, was 3–7 servings per week.

### 3.3. Optimized Food Pattern and Nutrient Intake

Table 3 shows the generated optimized food patterns in comparison with the actual dietary patterns for all food groups and subgroups in g/day, whereas Table 4 shows nutrient profiles for actual and optimized food patterns, clustered by the age group. Although variations have been clearly observed by child age brackets, the overall percentage of children meeting EAR or more for the selected nutrients from the actual feeding pattern was 54.3% for energy, 89.9% for protein, 68.1% for iron, 12.9% for zinc, 34.2% for calcium, 85.6% for vitamin A, and 79.7% for vitamin C. When the RDA level for target nutrients was computed and the percent coverage of each nutrient from the actual diet pattern was relatively lower for children in the younger age (6–11 months old). Based on the actual dietary pattern, percent coverage of protein and iron has met the nutrient requirement (RDA) for all target children.

Our result in Table 4 presents that the actual feeding pattern was not meeting the nutrient requirements (RDA), whereas the optimized dietary pattern has achieved nutrient adequacy for almost all the selected nutrients.

## 4. Discussion

The present study has identified the most common diet and nutrient adequacy potential of local diets that could be used for improving the nutrient intakes among infants and young children in limited resource settings.

Age-specific optimized food intakes that could meet the recommended dietary allowance (RDA) of target nutrients were achieved through the combination of locally available food items. However, such optimum RDA level for the target nutrients based on the local dietary pattern could not be successfully achieved without minimal consideration of locally accessible commercial foods. This suggests that a slight modification of the actual feeding pattern in realistic servings, minimally complimented with few commercial foods (such as baby-food cereals which are commonly consumed by 16.2% of children in the study area), has turned into adequate food intakes, substantially improving nutritional quality of the diet, and has achieved nutrient adequacy given the dietary reference intakes (DRIs) for children aged 6–23 months in developing countries.

Despite discrepancies between child age-brackets, most target nutrients (except, protein and iron) were found to be limiting nutrients. Of the nine target nutrients, zinc, vitamin A, vitamin C, and calcium were key problem nutrients, given the current dietary patterns in the study setting. Here, it is worth noting that zinc is the key problem nutrient, while iron is not given the current dietary pattern. Arguably, the likely reason for this could suggest the type of local foods frequently consumed (such as teff and mitin) which are good sources of iron. In addition, the nutritionally precooked and enriched baby-food cereals (e.g., Cerifam/Faffa) are accessible commercial foods in the study. The latter are also rich in minerals, particularly iron, iodine, and calcium phosphate.

Given the existing local dietary pattern, the percentage of children consuming EAR or more for most nutrients obtained from median size was 54.3%, 89.9%, 61.8%, 12.9%, 85.6%, 79.7%, and 34.2% for energy, protein, iron, zinc, vitamin A, vitamin C, and calcium, respectively. However, percentage of children in the early age-bracket (6–11 months of age) who were unable to meet the EAR for target nutrients from the usual diet was higher, as compared to older children (12–23 months old). For instance, percentage of RDA was 46.3% vs. 78.1% for zinc, 56.7% vs. 100% for vitamin A, 24.3% vs. 43.3% for vitamin C, and 40.0% vs. 50.0% for calcium in infants (6–11 months) and older children (11–23 months of age), respectively. However, careful combination of local foods has improved the nutrient intake in children across ages, achieving ≥99% RDA in the optimized diets.

The study setting was characterized by limited local diet diversity [11, 13], and this was also observed during our dietary records. Only 24 foods from a total of 68 food items recorded during our dietary records were consumed by ≥ 5% of the target population. However, the optimized food intake patterns were achieved within the range of lower (10th percentile) and upper (90th percentile) limits of actual intake patterns, demonstrating that the optimized diet is within the recorded cultural eating habits.

As expected, cereals and legumes were consumed more frequently as compared to animal source food in the study population. Cereal-based foods such as teff and bread were consumed not only most frequently but also relatively in larger quantities. Arguably, this finding may highlight the questionable result of high iron intake. Teff (*Eragrostis tef*) is a native cereal to Ethiopia and Eritrea, becoming globally popular due to the attractive nutritional profile such as gluten free and high dietary fiber content [33]. The attractive nutrients of teff include protein, dietary fiber, polyphenols, and certain minerals such as iron. And we could also point out that protein-rich foods, such as traditional legumes, when complemented with cereals, are sufficient to achieve full protein adequacy in children consuming plant-based diets, while substantially minimizing the question of any amino acid deficiency.

National reports [14] and other studies [34, 35] also confirm that homemade complementary foods are predominantly based on cereals and legumes and mostly an extension of family foods in Ethiopia. The dominant protein-source food items popularly consumed by children in the study setting were the home-based preparations of pea flour (stew/wot form), usually served together with other cereal products such as injera. The inclusion of such food items in dietary practice of children needs to be promoted, as this is found to be much cheaper than animal-source protein such as meat.

Previous study report [14] shows that consumption of animal-source foods as well as fruits and vegetables is very low at the national level. Also, our household-based dietary survey record has confirmed that fruits and vegetables rarely appeared in target population dietary patterns. In fact, even if there are favorable climatic conditions for diversified farming in the study setting, the production and consumption of fruits and vegetables were poor as compared to other field crops [36].

In addition and consistent with a previous study [5], mothers' (caregivers') knowledge about nutrition in decision-making when purchasing locally available food items, especially fruits and vegetables, is poor. Although access to fruits and vegetables is much more dictated by season, mothers' decision-making knowledge in food choice might have played a big role in limiting such important diet in the target population dietary system. Therefore, we suggest for community- (and/or household-) based nutrition education to encourage mothers/caregivers to consider including nutrient-dense foods including fruits and vegetables.

Generally speaking, the present analysis demonstrated that meeting nutritional goals for the target population requires an overall reduced consumption of grain, grain products, and condiment vegetables across ages. The LP process for the big food group (grain, grain products, roots, and tubers) needs to be decreased across ages. However, the situation is mostly different at the subgroup level. Teff and mitin flour, for example, are subgroups which were needed to be increased in the finally optimized diet across age bracket. Except for pea flour which was increased nearly by double, consumption of other protein-rich foods such as meats and eggs was reduced for infants aged 6–11 months. Limited local food diversity and the reduced choice of animal-source food options for our LP model match with the existing evidence [14] that diets of Ethiopian children often lack animal-source foods as well as fruits and vegetables. As a result, the actual homemade complementary foods are limited in micronutrients, such as iron, zinc, and calcium. However, iron intake shows not so poor despite previous findings, given the current dietary pattern.

Other local-based food preparations and consumptions which are assumed to be sources of protein and other nutrients are the likes of mitin flour, which is cereal-pulse blend in variable proportion. The amount of mitin flour in optimized food was needed to be increased across age, demonstrating the importance of this particular protein-rich local-based food preparation. However, this blend is individual home-based preparation, and the ratios of cereals-to-pulses were not yet properly standardized in the local context.

Our study has showed a huge gap pertinent to this particular local food item during analysis in associating with FCTs in ProPAN (2.0), and further details about it need to be explored in the future. Mitin flour preparation among households is not uniform, and it usually consists of many cereals and pulses (in different ratios) including but not limited to wheat, barley, emmer wheat, pea, and soybean. Also, a previous study has confirmed that [14] homemade complementary foods are frequently used in Ethiopia, and the basic recipe food items used for the preparation are based on locally available staples, while the choice of specific food item differs considerably between populations, owing to tradition, availability, and ease of access.

In poor communities such as Ethiopia, successfully identifying the true potential of local foods has comparative advantages over alternative strategies such as fortified commercial foods [14, 37]. Also, our market survey data show that the cost of commercial foods which are available in the local market such as baby-food cereals was comparable with meat. However, still cereal-based commercial foods such as Cerifam/Faffa and Mother's Choice were commonly consumed, especially among children in the early complementary period. This might be the reason that the amount of baby-food cereals in optimized food for infants aged 6–8 months was increased by more than double in the optimized diets.

The fact that appropriately designed complementary foods based on local resources are totally absent could be the possible explanation for such significant dependence of households on commercial foods to feed their young children in the study setting. Although commercial foods are relatively expensive than local foods, mothers (caregivers) opt to use commercial foods such as baby-food cereals because they think they are easy to prepare, safe (e.g., choking), less time-consuming, and/or demand minimal processing before served to children.

Evidence suggests that most infants are able to consume solid-consistency “family foods” by 12 months, even if they are still often served semisolid foods [14]. Therefore, for children in the early complementary period, home-based preparations of local food items are time-consuming and may also cause choking if the size, shape, and consistency of food are not carefully managed. Whatever the case is, the use and emphases on the appropriate formulation of homemade complementary foods remain best solutions to address the lack of appropriately prepared child diet in the study setting.

Interestingly, the percentage of children who received cow milk the day before the survey was quite significant. In Ethiopia, fresh cow milk is locally produced and mainly given to small children [35]. Our analysis has identified a correlation between cow milk and commercial baby-food cereal consumptions when stratified by child age. The optimized diet needs the amount of cow milk and baby-food cereals to be increased only among children in the early age-bracket. The challenges of dietary diversity of local diet might have forced our LP process to consider increased consumption of cow milk and commercial foods to achieve nutrient adequacy among the early aged-bracket. Although the availability of cow milk in a sustainable manner is a challenge [38], milk is known for its significant proportion of nutrients [31, 38], such as high-quality protein and micronutrients. The availability of cow milk in the local dietary patterns and its inclusion in our LP process could easily influence results. However, we found it is necessary to encourage the milk production and consumption among children to improve the nutritional status, even though it might be impractical for lower-income families to get access.

As described earlier, protein and iron were not problem nutrients in the local dietary system of the target population. Pea flour and other legumes, which are frequently available in the local diets, are obviously rich in protein [31], and they could also have a high content of other micronutrients. Other nutrient-dense foods in the local diets such as milk, eggs, and meats were also not ignored here in helping ensure the adequate intake of protein and iron. As a result, optimal requirement of these nutrients (protein and iron) was achieved for the target children, even when the actual dietary practices would be maintained.

Evidence shows that, with elimination of meat and increased intake of phytate-containing legumes (such as pea) and whole grains, the absorption of both iron and zinc is lower in plant-based diets [39, 40]. However, household processing such as heat treatment, sprouting, fermentation, and malting and the use of ideal combinations of food components can significantly improve micronutrient bioavailability of iron and *β*-carotene from plant foods [40]. In the present study, it is important to note that given the current dietary pattern, most of the house-based food preparations (such as pea flour, mitin, and cereal grains) are usually given to children after prudent cooking in the study area, arguably suggesting the possible reason for meeting iron requirement, while this was not the case for zinc. Thus, the author would like to encourage the use of ideal combinations of food components and also the maximum use of nutrient-dense food items such as milk, eggs, and meats to address problem nutrients in the area.

Moreover, vitamin A (*µ*g of RAE/day) requirements could also be ensured with actual dietary practice, but only among older children (12–23 months). However, the percent coverage for vitamin A below RDA was 43.3% among infants (6–11 months). The local dietary pattern which relies on higher servings of grain and legumes and inclusion of cow milk, meat, and eggs which are rich in protein, in turn, also provide sufficient amount of other nutrients such as iron and vitamin A.

Another interesting point worth mentioning is that we could not totally rule out the moderate bioavailability level chosen for our analysis which might have overestimated iron adequacy in the actual diet. Given the existing local dietary pattern, zinc, vitamin C, and calcium were found adequate across age. Percent coverage below RDA for zinc, vitamin C, and calcium was 53.7%, 75.7%, and 60.1% in the target population, respectively.

However, in the present study, the best solution was identified through careful combination of local foods at the cost of 20.50, 21.80, and 18.60 Ethiopian birr per day (∼0.7 USD/day) for children aged 6–8, 9–11, and 12–23 months, respectively. The optimized combination of local diet was found to be possible at reasonable cost per day as compared to the actual dietary pattern, though this might not necessarily suggest affordability of the optimized diet for the entire population.

Despite identified strengths, some limitations inherent to dietary and LP analysis are acknowledged. LP of NutriSurvey for Windows is a useful tool for identifying and analyzing whether locally available foods could provide the nutrients needed by young children. It also helps to decide the quantity of nutrient-rich foods needed to provide optimal nutrients at reasonable cost. However, the outcome of the LP process relies on many parameters such as the quality of the dietary data collected, the FCTs used, and assumed bioavailability of some nutrients.

In the present study, data were collected during the rainy preharvest season, and results might not be necessarily extrapolated to other seasons. Dietary and seasonal variations in food availability and intakes are needed to be considered when designing dietary recommendations based on local foods in the study setting. Moreover, the nutrition profile of some foods was either missing or limited in FCTs for use in Ethiopia [23]. As a result, we were forced to use food databases from other countries including FCTs of the USA [24] and other East African countries [25, 26]. However, we have also tried to avoid overdependence on other countries' food composition databases by analyzing limited nutrients where nutritional profiles were fully available.

In addition, during the LP process, we only selected most commonly consumed foods by ≥ 5% children, with data from only 24 food items considered in the model. Therefore, we are not sure if including foods consumed by few children might have changed the solution, influencing our results. Including less consumed foods into the model may limit the options that LP analysis has for selection of foods such as fruits and vegetables, resulting in more problem nutrients outside our target nutrients than expected.

## 5. Conclusion

Arguably, our analysis has demonstrated the potential of the local dietary pattern in developing improved combination of food-based dietary recommendation in the target population. Careful modifications of the given feeding pattern in realistic servings, slightly complimented with locally accessible commercial foods, have turned into adequate food intakes (met the RDA level) for target nutrients, while maintaining medium sizes of the actual dietary pattern and livelihood, given the dietary reference intakes (DRIs) for infants and young children aged 6–23 months in developing countries.

## Figures and Tables

**Table 1:** Nutritional status of children aged 6–23 months in urban/periurban slums of west Oromia, Ethiopia^*a*^.

Characteristics (*n *= 396)	Mean	SD
*Background*		
Children's weight in kg (overall)	9.62	1.70
Infants (6–11 months of age)	8.95	1.42
Young children (12–23 months of age)	10.33	1.69
Children's height/length in cm (overall)	73.57	5.13
Infants (6–11 months of age)	70.48	3.35
Young children (12–23 months of age)	76.85	4.64
Children's weight in months (overall)	12.39	4.96
Infants (6–11 months of age)	8.44	1.74
Young children (12–23 months of age)	16.59	3.63

*Nutritional status of IYC* ^*b*^		
Height-for-age, *Z*-score (HAZ)	−0.67	1.41
Children being stunted, *n* (%)	60 (15.2)	
Weight-for-age, *Z*-score (WAZ)	0.12	1.26
Children being underweight, *n* (%)	20 (5.1)	
Weight-for-height, *Z*-score (WHZ)	0.62	1.53
Children being wasted, *n* (%)	23 (5.8)	

*Energy (kcal) consumed in a 24-hour period* ^*c*^		
Overall, age (*n* = 396)	529.27	434.05
Infants (6–11 months of age) (*n* = 204)	369.42	307.36
Young children (12–23 months of age) (*n* = 192)	699.11	482.62

^*a*^Values are mean standard deviation, unless stated otherwise. ^*b*^Anthropometric *Z*-scores -2 SD were considered to categorize IYC based on the WHO growth standards. ^*c*^Energy (kcal/day) intake of IYC from the median actual dietary pattern.

**Table 2:** Actual patterns and cost constraints included in NutriSurvey linear programming^*a*^.

Food groups and subgroups (g/day)	Consumed by percentage of children	Serving size (g/day)^*c*^									Servings/week^*d (e)*^	Price/100 g, edible port.
		6–8 months (*n* = 103)			9–12 months (*n* = 101)			12–23 months (*n* = 196)				
		Min.	Med.	Max.	Min.	Med.	Max.	Min.	Med.	Max.		
Grain, grain products, roots, and tubers		111.4	214.4	633.8	125.2	239.6	712.1	138.0	391.8	940.0		
Teff, cooked *(injera)*	67.9	8.5	20.0	30.0	11.0	23.0	57.0	12.0	30.0	63.0	3 (4)	1.00
Bread	22.0	21.0	22.0	152.0	13.0	31.0	76.0	20.0	44.0	103.0	0 (7)	1.00
Baby food, cereals^*b*^	16.2	7.0	11.5	25.0	6.0	12.0	27.0	8.0	13.5	39.0	0 (7)	18.00
Cereal-pulse blend (mitin flour)	15.2	4.0	15.0	28.0	8.0	14.0	30.0	9.0	25.5	40.0	0 (5)	4.00
Irish potatoes, w/skin, raw	14.6	19.0	38.0	158.0	10.0	17.0	138.0	11.0	41.5	147.0	0 (4)	1.00
Spaghetti, wheat, boiled	14.1	3.8	32.3	71.9	15.4	25.0	69.2	16.2	37.3	100.0	0 (4)	2.80
Emmer wheat, flour (soup/gruel)	12.9	8.0	15.5	38.0	5.0	15.0	37.0	9.0	24.5	65.5	0 (4)	2.00
Irish potatoes, boiled (wo/skin)	11.6	12.0	22.0	39.0	29.0	53.0	86.0	20.0	75.5	111.0	0 (4)	1.00
Macaroni, wheat, boiled	8.8	0.0	0.0	0.0	12.2	23.1	125.0	24.2	58.1	146.5	0 (5)	2.00
Rice, white, boiled	7.6	28.1	38.1	91.9	15.6	26.5	66.9	8.6	41.9	125.0	0 (4)	5.00

Legumes, nuts, and seeds												
Peas flour (stew/wot)	50.0	1.5	4.2	10.4	3.1	5.0	16.5	1.5	7.7	16.2	3 (4)	3.00

Meats, fish, and eggs		**35.0**	**64.0**	**86.0**	**54.5**	**89.5**	**163.5**	**33.5**	**108.5**	**187.0**		
Chicken egg, fried w/fat	19.4	15.0	31.0	45.0	18.5	32.0	54.5	22.5	41.5	68.0	0 (4)	6.00
Meat	6.6	11.0	22.0	24.0	26.0	31.0	63.0	4.0	30.0	61.0	0 (3)	18.00
Chicken egg, hard-boiled	6.3	9.0	11.0	17.0	10.0	26.5	46.0	9.0	37.0	58.0	0 (4)	7.00

Dairy products												
Milk, cow	36.6	41.0	115.0	291.0	74.0	160.0	342.0	82.0	160.0	428.0	0 (4)	2.00

Vegetables		**24.0**	**54.0**	**154.5**	**15.0**	**53.0**	**123.0**	**8.0**	**65.0**	**158.0**		
Onion, fresh, raw	56.3	6.0	12.0	34.0	2.0	15.0	39.0	4.0	17.0	42.0	3 (5)	1.00
Garlic, fresh, raw	27.8	1.0	1.0	3.5	1.0	1.0	5.0	1.0	2.0	7.0	0 (4)	5.00
Tomatoes, red, raw	12.1	17.0	41.0	117.0	12.0	37.0	79.0	3.0	46.0	109.0	0 (5)	1.00

Beverages												
Black tea, w/sugar added	13.4	34.0	72.0	110.0	19.0	44.5	121.0	22.0	67.0	140.0	0 (5)	9.00

Miscellaneous		**5.0**	**10.0**	**28.0**	**6.0**	**14.5**	**40.0**	**7.0**	**16.0**	**59.0**		
Table salt (and/or iodized salt)	83.1	1.0	1.0	2.0	1.0	1.0	2.0	1.0	1.0	3.0	4 (5)	0.10
Butter (wo/salt, spiced)	67.2	1.0	1.0	7.0	1.0	2.0	7.0	1.0	3.0	10.0	3 (4)	17.00
Palm oil, red	61.4	1.0	3.0	10.0	1.0	3.0	8.0	1.0	3.0	11.0	3 (4)	3.00
Sugar	48.5	1.0	4.0	8.0	2.0	7.5	21.0	3.0	8.0	33.0	3 (4)	2.00
Spices, chili powder	18.4	1.0	1.0	1.0	1.0	1.0	2.0	1.0	1.0	2.0	0 (4)	6.00

^*a*^Food items consumed by ≥ 5% of children (*n* = 396). ^*b*^Commercial baby-food cereals particularly included in this subgroup are Cerifam (Faffa) and Mother's Choice. ^*c*^Values are the minimum, median, and maximum serving sizes of raw edible portions when consumed in the 24-hour period, calculated on the basis of the 10th, 50th, and 90th percentile, respectively. ^*d*^Values are the median number of servings calculated on the basis of the 50th percentile, when considering all target children (*n* = 396). ^*e*^Values are the median number of servings calculated on the basis of the 50th percentile, when considering those children who reported ever consuming food.

**Table 3:** Comparison of food amount (g/day) between actual and optimized food intakes.

Food groups and subgroups (g/day)^*a*^	Consumed by percentage of children	Serving size (g/day)					
		6–8 months (*n* = 103)		9–12 months (*n* = 101)		12–23 months (*n* = 196)	
		Actual	Optimized	Actual	Optimized	Actual	Optimized
Grain, grain products, roots, and tubers		**214.4**	**154.0**	**239.6**	**198.0**	**391.8**	**284.0**
Teff, cooked (injera)	67.9	20.0	9.0	23.0	57.0	30.0	63.0
Bread	22.0	22.0	21.0	31.0	18.0	44.0	84.0
Baby food, cereals	16.2	11.5	25.0	12.0	6.0	13.5	8.0
Cereal-pulse blend (mitin flour)	15.2	15.0	28.0	14.0	30.0	25.5	40.0
Irish potatoes, w/skin, raw	14.6	38.0	19.0	17.0	10.0	41.5	11.0
Spaghetti, wheat, boiled	14.1	32.3	4.0	25.0	15.0	37.3	16.0
Emmer wheat, flour, raw	12.9	15.5	8.0	15.0	5.0	24.5	9.0
Irish potatoes, boiled, wo/skin	11.6	22.0	12.0	53.0	29.0	75.5	20.0
Macaroni, wheat, boiled	8.8	0.0	0.0	23.1	12.0	58.1	24.0
Rice, white, boiled	7.6	38.1	28.0	26.5	16.0	41.9	9.0

Legumes, nuts, and seeds							
Peas flour, raw	50.0	4.2	8.0	5.0	3.0	7.7	2.0

Meats, fish, and eggs		**64.0**	**35.0**	**89.5**	**55.0**	**108.5**	**36.0**
Chicken egg, fried w/fat	19.4	31.0	15.0	32.0	19.0	41.5	23.0
Meat	6.6	22.0	11.0	31.0	26.0	30.0	4.0
Chicken egg, hard-boiled	6.3	11.0	9.0	26.5	10.0	37.0	9.0

Dairy products							
Milk, cow	36.6	115.0	208.0	160.0	206.0	160.0	133.0

Vegetables		**54.0**	**24.0**	**53.0**	**15.0**	**65.0**	**8.0**
Onion, fresh, raw	56.3	12.0	6.0	15.0	2.0	17.0	4.0
Garlic, fresh, raw	27.8	1.0	1.0	1.0	1.0	2.0	1.0
Tomatoes, red, raw	12.1	41.0	17.0	37.0	12.0	46.0	3.0

Beverages							
Black tea, w/sugar added	13.4	72.0	34.0	44.5	19.0	67.0	22.0

Miscellaneous		**10.0**	**8.0**	**14.5**	**10.0**	**16.0**	**18.0**
Table salt (and/or iodized salt)^*b*^	83.1	1.0	1.0	1.0	1.0	1.0	1.0
Butter (wo/salt, spiced)	67.2	1.0	1.0	2.0	1.0	3.0	1.0
Palm oil, red	61.4	3.0	4.0	3.0	3.0	3.0	9.0
Sugar^*c*^	48.5	4.0	1.0	7.5	2.0	8.0	3.0
Spices, chili powder^*b*^	18.4	1.0	1.0	1.0	1.0	1.0	1.0

Price (local cost/day)		31.43	20.50	30.99	21.80	41.04	18.60

^*a*^Optimization based on the raw edible portions when consumed in the 24-hour period, calculated on the basis of the 10th, 50th, and 90th percentile, respectively. ^*b*^Upper limits (maximum) for LP function were set at the 50th percentile.

**Table 4:** Comparison of energy and nutrient adequacy between actual and optimized dietary patterns for IYC in west Oromia (Ethiopia).

Nutrients	Percentage of children meeting^*a*^ ≥100% EAR			Actual nutrient intake level (RDA)^*b*^			Percentage of RDA			Nutrient contents of optimized diet (≥99% RDA)		
	6–23 mos	6–11 mos	12–23 mos	6–23 mos	6–11 mos	12–23 mos	6–8 mos	9–11 mos	12–23 mos	6–23 mos	6–11 mos	12–23 mos
Energy (kcal/day)^*c*^	54.3	60.8	47.4	429.8	310.2	593.9	50.4	45.2	66.4	622.1	690.8	891.0
Protein (g)	89.9	86.7	93.2	18.0	13.5	23.8	100.0	100.0	100.0	23.3	27.6	28.1
Fat (g), 30%^*c*^	*n/a*	*n/a*	*n/a*	17.3	13.5	23.8	65.9	59.0	86.6	25.2	25.7	29.6
CHO (g), 51%	*n/a*	*n/a*	*n/a*	53.1	40.1	76.4	*n/a*	*n/a*	*n/a*	79.5	91.4	133.8
Iron (mg/day)^*d*^	68.1	45.8	91.7	13.3	9.8	18.0	100.0	100.0	100.0	13.3	18.1	20.5
Zinc (mg/day)^*d*^	12.9	10.8	15.1	2.4	1.9	3.2	46.3	46.3	78.1	4.2	5.2	5.9
Vitamin A (*μ*g of RAE/day)	85.6	85.2	85.9	328.2	226.7	448.8	56.7	56.7	100.0	412.5	411.3	603.5
Vitamin C (mg/day)	79.7	99.5	58.9	10.1	7.3	13.0	24.3	24.3	43.3	44.3	46.0	48.8
Calcium (mg/day)	34.2	22.7	46.4	179.5	159.5	199.8	40.0	40.0	50.0	400.7	400.0	399.9

^*a*^Percentage of children consuming 100% or more of the daily median energy and nutrient intake recommendation (median means if 50% or more of the children meet the recommendation), IOM [28]. IOM conversion factors of 1.2 for zinc, vitamin C, and calcium and 1.4 for vitamin A were used. For protein and iron, 66% of the RDI was used as a proxy for the EAR, as previously recommended [26]. Adequate nutrient intake of the children sampled was assumed if 100% of the children meet the proxy EAR. ^*b*^Nutrient intake level (RDA) calculated from a median serving size in actual dietary patterns, based on the FAO and WHO (2002) recommended nutrient intakes [30]. ^*c*^Nutrient intake level (RDA) calculated from the EAR based on a median serving size in actual dietary patterns. ^*d*^Assuming medium iron and zinc bioavailability as recommended by the FAO and WHO (2002) [30]. *n/a*: values are not calculated, and only their overall percentage contributions to the total energy were indicated. RAE: retinol activity equivalent.

## Data Availability

The datasets used and/or analyzed during the current study are available from the corresponding author on reasonable request.
